# Differential Stability of Cell-Free Circulating microRNAs: Implications for Their Utilization as Biomarkers

**DOI:** 10.1371/journal.pone.0075184

**Published:** 2013-09-20

**Authors:** Verena Köberle, Thomas Pleli, Christian Schmithals, Eduardo Augusto Alonso, Jörg Haupenthal, Halvard Bönig, Jan Peveling-Oberhag, Ricardo M. Biondi, Stefan Zeuzem, Bernd Kronenberger, Oliver Waidmann, Albrecht Piiper

**Affiliations:** 1 Department of Medicine I, University Hospital Frankfurt, Frankfurt, Germany; 2 German Red Cross Blood Service Baden-Württemberg-Hessen, Institute for Transfusion Medicine and Immunohematology of the University Hospital Frankfurt, Frankfurt, Germany; The University of Queensland, Australia

## Abstract

**Background:**

MicroRNAs circulating in the blood, stabilized by complexation with proteins and/or additionally by encapsulation in lipid vesicles, are currently being evaluated as biomarkers. The consequences of their differential association with lipids/vesicles for their stability and use as biomarkers are largely unexplored and are subject of the present study.

**Methods:**

The levels of a set of selected microRNAs were determined by quantitative reverse-transcription PCR after extraction from sera or vesicle- and non-vesicle fractions prepared from sera. The stability of these microRNAs after incubation with RNase A or RNase inhibitor, an inhibitor of RNase A family enzymes was studied.

**Results:**

The levels of microRNA-1 and microRNA-122, but not those of microRNA-16, microRNA-21 and microRNA-142-3p, declined significantly during a 5-h incubation of the sera. RNase inhibitor prevented the loss of microRNAs in serum as well as the degradation of microRNA-122, a microRNA not expressed in blood cells, in whole blood. Stabilization of microRNA-122 was also achieved by hemolysis. Prolonged incubation of the sera led to enrichment of vesicle-associated relative to non-vesicle-associated microRNAs. Vesicle-associated microRNAs were more resistant to RNase A treatment than the respective microRNAs not associated with vesicles.

**Conclusions:**

Serum microRNAs showed differential stability upon prolonged incubation. RNase inhibitor might be useful to robustly preserve the pattern of cell-free circulating microRNAs. In the case of microRNAs not expressed in blood cells this can also be achieved by hemolysis. Vesicle-associated microRNAs appeared to be more stable than those not associated with vesicles, which might be useful to disclose additional biomarker properties of miRNAs.

## Introduction

MicroRNAs (miRNAs) are short non-coding regulatory RNAs that mediate posttranscriptional gene regulation by binding to and repressing specific messenger RNA targets [1]. Up to now more than 1,700 miRNAs have been identified (www.mirbase.org). Abnormal cells or tissues show dysregulated miRNA expression pattern [2].

miRNAs are also present in the circulation in a cell-free form, showing remarkable stability despite high circulating RNase activity [3-5]. Extracellularly circulating miRNAs are either associated with different types of vesicles or lipoprotein complexes, including exosomes, microvesicles, apoptotic bodies and lipoproteins [6-9] or are complexed with proteins such as Ago2 apparently without being encapsulated into vesicles [6,7]. Nevertheless, there is no consensus on the relative contribution of vesicle-associated miRNAs to the total miRNA content in sera from healthy donors [10].

The association of cell-free circulating miRNA with lipids/vesicles confers targeting properties to the miRNAs. Upon delivery to target cells, vesicle-associated miRNAs can alter the behaviour of the target cells [8,11]. Another important aspect of extracellularly circulating miRNAs is that their profiles in blood serum or plasma are altered in patients [3-5], indicating that cell-free circulating miRNAs might be highly useful as new blood-based disease markers. In particular, elevated levels of tissue-specific miRNAs in the blood circulation have potential as clinically useful disease markers. For instance, elevated levels of miR-122, which is almost exclusively expressed in the liver [12], in blood serum or plasma are highly specific and sensitive marker for liver damage [13-15]. They reflect the response to therapy in patients with chronic hepatitis C [16] and are prognostic in patients with liver cirrhosis [17] and in patients with hepatocellular carcinoma [18]. Together, these studies indicate that extracellularly circulating miRNAs can supplement the current set of diagnostic and prognostic markers, thereby improving their diagnostic power.

The association of cell-free circulating miRNA with lipids/vesicles is likely to reflect different physiological or pathophysiological processes. In favor of this, a recent study in mice reported that toxic liver injury caused an increase of a miR-122 pool in plasma, which did not precipitate with the exosomal fraction, whereas CpG-induced hepatitis was accompanied by elevated levels of the exosomal pool of miR-122 [19]. Therefore, information on whether abnormal levels of serum or plasma miRNAs are due to changes in the vesicle- or the non-vesicle-associated pool is likely to provide diagnostic information exceeding that provided by the levels of unfractionated serum or plasma miRNAs. Nevertheless, the impact of the differential association of miRNAs with lipids/vesicles on their stability, their use as biomarkers and functional studies is still poorely defined. Here we report that serum miRNAs differed in their stability. miRNAs associated with vesicles appeared to be more stable and more resistant to RNase A treatment than serum miRNAs not associated with vesicles. Our data further indicate that stabilization of extracellularly circulating miRNAs not expressed in blood cells can be achieved by hemolysis. Inhibition of RNase A family enzymes or hemolysis might thus be useful to preserve cell-free circulating miRNAs, whereas RNase A treatment might be used as fast and convenient method to enrich vesicle-associated miRNAs.

## Methods

### Blood sampling

Peripheral blood was collected from healthy donors. Written consent was obtained from all subjects in accordance with the Declaration of Helsinki guidelines, and with approval from the Ethics Committee of the University Hospital, Frankfurt.

Eight mL of blood was collected from each individual directly into serum collection tubes (no. 02.1063, Sarstedt). The blood was centrifuged at 1500 *g* for 10 min at 4°C. The supernatant was transferred to Eppendorf tubes and additionally centrifuged at 2000 *g* for 3 min to completely remove any remaining cells. In some experiments, RNase inhibitor (RI) (N8080119, Life Technologies), which inhibits type A RNases (RNase A, B and C), was added to the blood tubes prior to the blood drawing. Prior to the addition to the blood tubes, the buffer of the RI was replaced with PBS by gel filtration through PD SpinTrap G-25 columns (GE Healthcare).

### Preparation of erythrocytes

Whole blood was collected from healthy volunteer donors into Composelect bags (Fresenius), component separated using the Compomat device (Fresenius). An erythrocyte concentrate was generated by passing the erythrocyte-rich fraction through a leuko-depletion filter into SAG-M buffer. An anonymized tube segment from a fresh erythrocyte concentrate was removed for these studies. The content (purity >99.9%) was washed twice and resuspended in PBS. Use of the cells was with informed consent of the donor and permission by the Ethics Committee of the Goethe University.

### Preparation of vesicle and non-vesicle fractions

Freshly prepared serum was centrifuged at 10,000 *g* for 30 min at 4°C to remove any cell fragments. Thereof, 800 µL were diluted with 3.2 mL of PBS solution and mixed gently. Samples were centrifuged in 4-mL (11 × 60 mm) polyallomer tubes (no. 328874; Beckman Coulter) at 120,000 *g* for 120 min at 4°C in a swinging-bucket rotor. A 200 µL aliquot of the supernatant was used for RNA isolation. Vesicle pellets were resuspended in 200 µL PBS solution. The fractions were analyzed for the exosomal marker CD81 by immunoblotting under non-reducing conditions as described recently [20], using Huh-7 cell lysate as positive control. As shown in Figure S1, CD81 immunoreactivity was enriched in the vesicle fraction.

### miRNAs analysis

RNA was extracted from 200 µL of serum or 200 µL of the supernatant or pellet (after resuspending in PBS) fraction with the miRNeasy Mini Kit (Qiagen) according to the recommendations of the manufacturer. RNA was eluted in 30 µL RNase-free water. RNA was reverse transcribed using the TaqMan microRNA reverse transcription kit and TaqMan assays (Life Technologies). To standardize the reverse transcription reaction a volume of 5 µL RNA was reverse transcribed. Real-time PCR was performed in duplicates with 3 µL of cDNA using the TaqMan Universal Master Mix II, no UNG (Life Technologies) and TaqMan assays for a selected set of miRNAs, which had been associated with liver diseases, on a StepOne Plus instrument. PCR was carried out as recommended by the manufacturer. Data were processed with the StepOne software (version 2.0).

### Statistical analysis

Unless otherwise stated, all experiments were performed at least on three different occasions with similar results. The values are given as means ± standard deviation (SD). Relative miRNA levels were compared between different treatment groups with the Kruskal-Wallis test or the Wilcoxon-Mann-Whitney-U test using the BiAS software for Windows (version 10.02, Epsilon publishing).

## Results

### miRNAs show different kinetics of decline in serum

**Figure 1:**
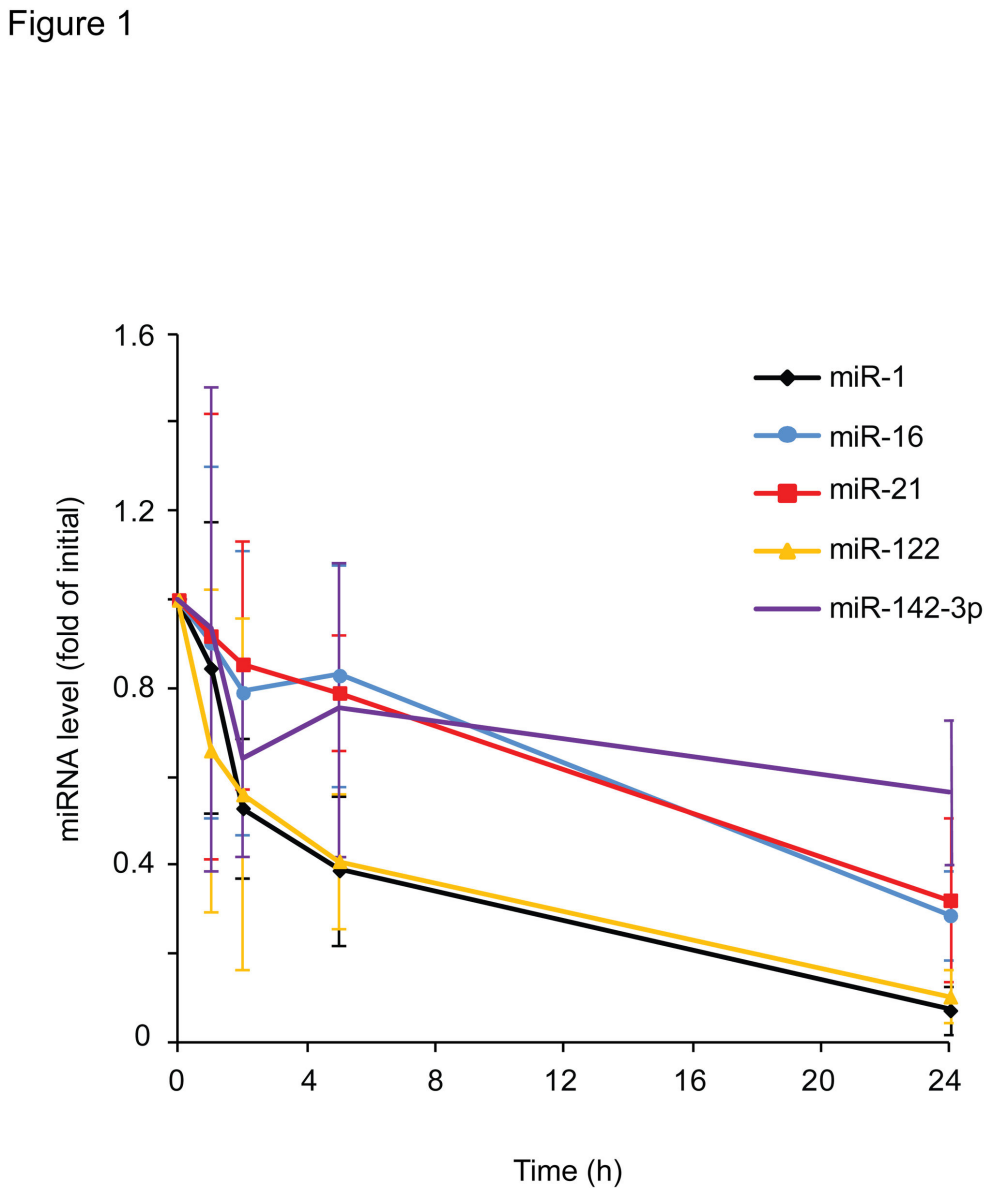
The levels of miRNAs decline with different kinetics in serum. Serum (n ≥ 3) was separated from blood cells directly after blood drawing and incubated at room temperature for different durations (0, 1, 3, 5 or 24 h) before extraction of the RNA. Levels of five different microRNAs (miR-1, miR-16, miR-21, miR-122 and miR-142-3p) were analyzed by reverse transcription real-time PCR and normalized to their initial values. To test for statistically significant differences between the five microRNAs a Kruskal-Wallis test with multiple Dunn comparison and Bonferroni-Holm correction was performed. This revealed significant differences between miR-16 and miR-122(P < 0.001) and miR-21 and miR-122(P < 0.01).

Overall, endogenous miRNAs have been found to be stable in blood serum or plasma [3-7]. However, we hypothesized that the stability of individual miRNAs might vary. To test this sera from healthy subjects were incubated for 0, 1, 3, 5 or 24 h at room temperature. Subsequently, total RNA was isolated from the sera and the levels of miR-1, miR-16, miR-21, miR-122 and miR-142-3p were determined as marker miRNAs by reverse transcription PCR. As shown in Figure 1, the levels of miR-1 and miR-122 declined substantially within 3 h of incubation, whereas the levels of miR-16, miR-21 and miR-142-3p declined only slightly within the initial 5 h of incubation. These data indicate that endogenous serum miRNAs differ in their stability.

### RNase inhibitor (RI) reduces the loss of miRNAs from serum

**Figure 2:**
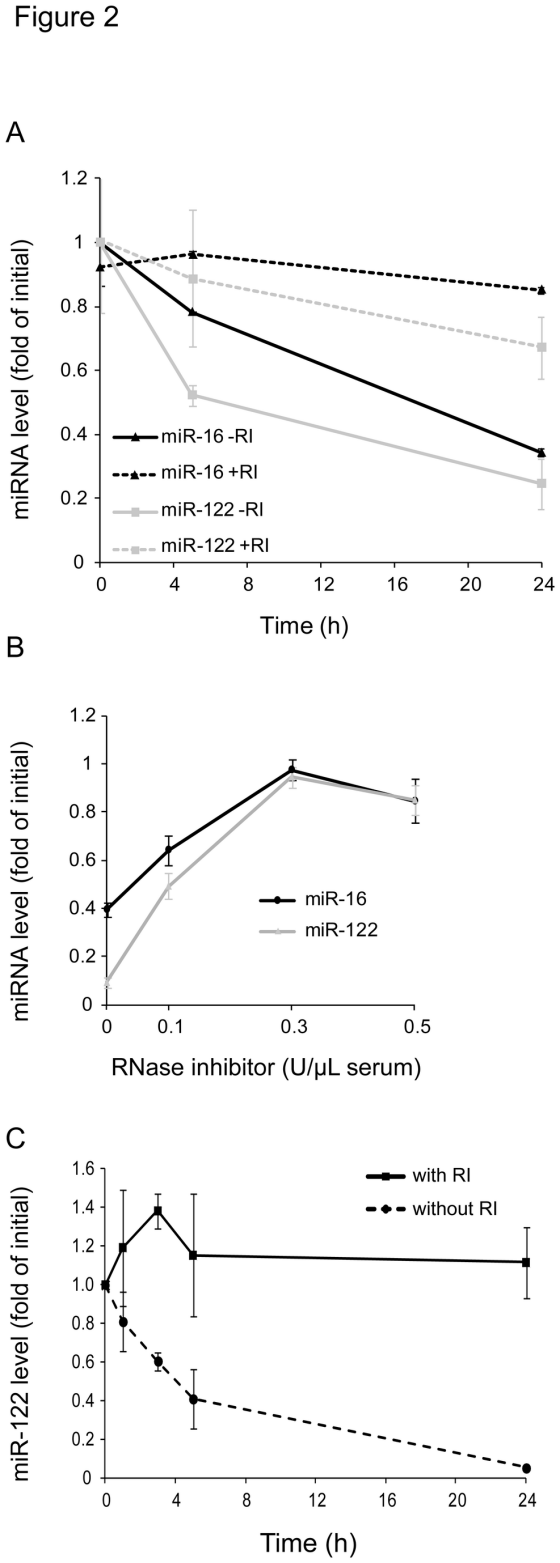
RNase inhibitor (RI) reduces the loss of miRNAs from serum and whole blood. (A) Serum was incubated for 0, 5 or 24 h with or without RI (0.2 U/µL serum) before RNA isolation. Levels of miR-16 and miR-122 were analyzed by reverse transcription real-time PCR and normalized to the initial value (0 h). A representative figure from three independent experiments with similar results is displayed. (B) Serum was incubated for 24 h with different concentrations of RI (0, 0.1, 0.3 or 0.5 U/µL serum), miR-16 and miR-122 levels were analyzed and normalized to the corresponding levels without serum incubation. The data shown is representative for three independent experiments with similar results. (C) Whole blood in serum tubes (n = 3) was incubated with and without RI (0.3 U/µL blood) for 0, 1, 3, 5 or 24 h at room temperature, followed by separation of serum from blood cells. RNA was isolated from 200 µL of serum and miR-122 levels were analyzed by reverse transcription real-time PCR. MicroRNA levels were normalized to the initial value at timepoint 0. Wilcoxon-Mann-Whitney-U test revealed statistical significance (P < 0.001) between miR-122 serum levels with or without RI.

RI inhibits enzymes of the RNase A family, among which human pancreatic RNase accounts for approximately 70% of RNase activity contained in human serum [21-23]. To investigate if RI could be used to better preserve the miRNA pattern in blood serum, sera were incubated with 0.2 U/µL of RI for different duration, followed by the determination of the levels of miR-16 and the labile miR-122 in the sera. As illustrated in Figure 2A, RI attenuated the decline of miR-16 and miR-122. Examination of the dose-response curve for the effect of RI on the levels of these miRNAs revealed that 0.1 U/µL already inhibited the loss of miR-16 and miR-122 during an incubation for 24 h (Figure 2B). 0.3 U/µL RI almost completely prevented the loss of these miRNAs from the sera.

To investigate if RI also prevents the loss of labile extracellular miRNAs in whole blood, RI was added to the blood tubes prior to the drawing of the blood, followed by incubation of the blood tubes for different times and preparation of sera and extraction of the RNA. To avoid alterations by miRNAs released from blood cells during the incubation, the liver-specific miR-122 was selected in these analyses. As shown in Figure 2C, RI almost completely prevented the loss of miR-122, suggesting that RI could also prevent the degradation of extracellular miRNA in whole blood.

### Hemolysis improves the stability of circulating microRNAs

**Figure 3:**
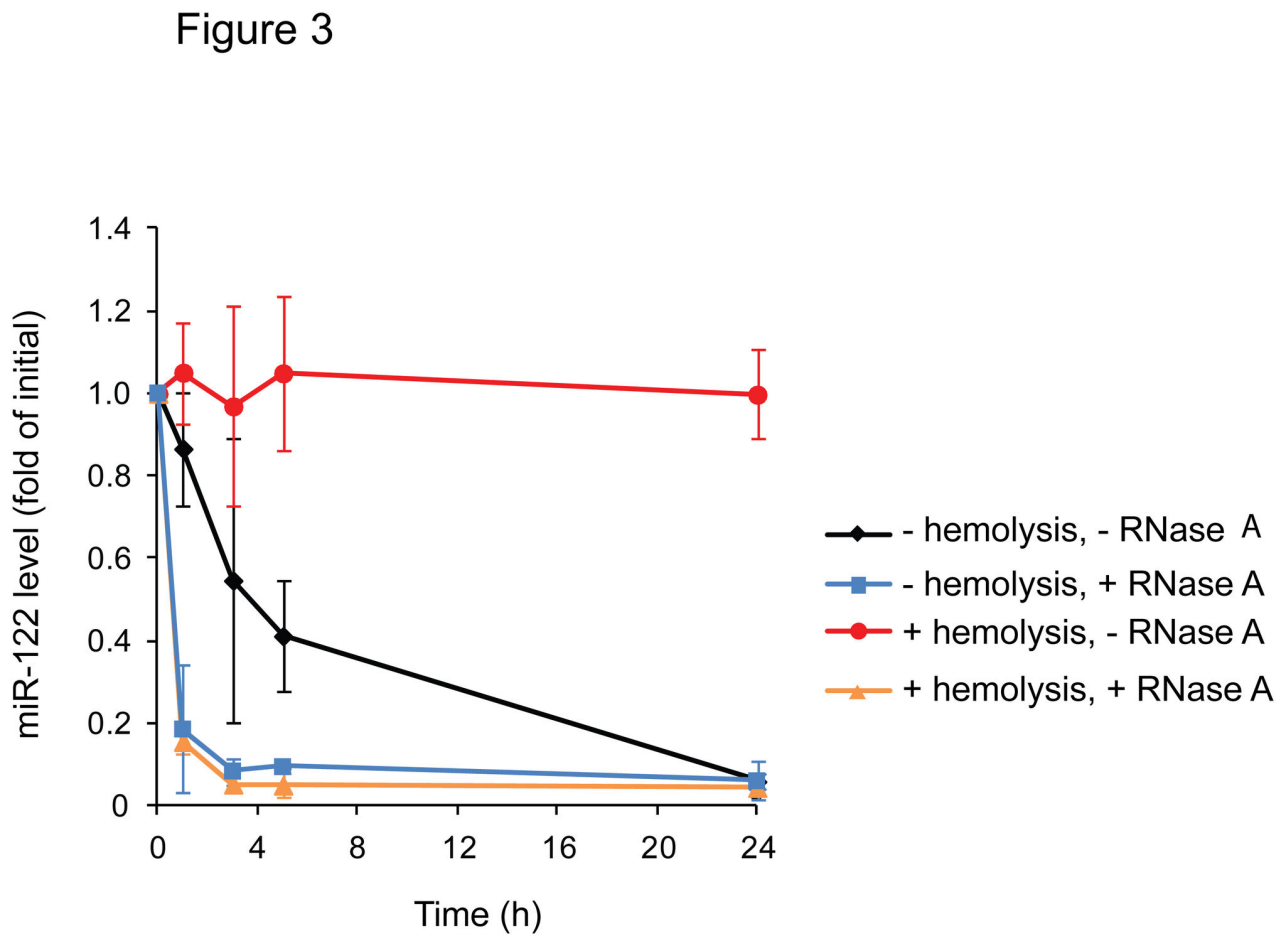
Hemolysis reduces the degradation of miR-122. Blood was drawn from three healthy volunteers into serum tubes prior to the addition of Triton X-100 (final concentration 0.07%) or PBS (control) and RNase A (final concentration 10 U/mL) or PBS. Sera were separated from blood cells and were incubated at room temperature for different periods (0, 1, 3, 5, 24 h) prior to RNA isolation and analysis of miR-122 levels. Kruskal-Wallis test with multiple Dunn comparison and Bonferroni-Holm correction revealed statistically significant differences between miR-122 levels with or without hemolysis without RNase A treatment (P < 0.05) and between miR-122 levels under hemolytic conditions with or without RNase A treatment (P < 0.001). RNase A treated samples with or without hemolysis showed no significant differences.

miR-122 is almost exclusively expressed in the liver and its extracellularly circulating levels are a new promising highly specific marker of liver damage; it may also serve as prognostic marker in patients with chronic liver disease [13-19]. In agreement with the assumption that miR-122 is not expressed in blood cells, hemolysis had no significant effect on serum miR-122 levels, whereas it led to dramatic increases in the concentrations of the ubiquitously expressed miR-16 and miR-21 by several orders of magnitude (data not shown). To investigate if hemolysis alters the stability of serum miR-122, the blood samples lysed with Triton X-100 or not (control with PBS) were incubated for 0, 1, 3, 5 or 24 h at room temperature, followed by quantification of cell-free miR-122. As illustrated in Figure 3, the levels of miR-122 in hemolysed samples showed no significant decline within the observation period, whereas in the sera from the control samples miR-122 levels declined significantly after 5 h of incubation. Thus, hemolysis prevented the loss of miR-122 from the blood samples. Nevertheless, upon addition of RNase A to hemolysed blood samples miR-122 levels rapidly declined (Figure 3).

**Figure 4:**
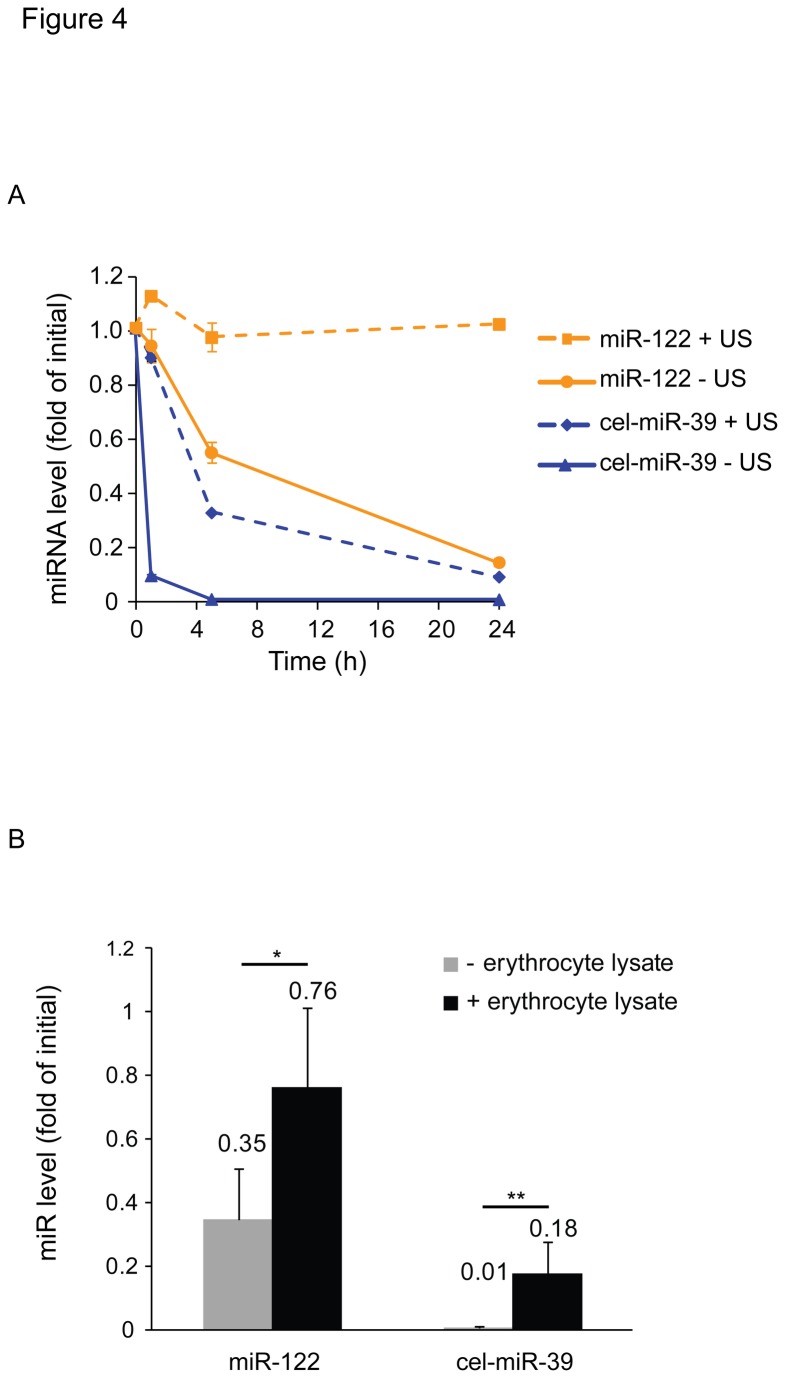
Release of RI from blood cells prevents loss of miR-122 and exogenous cel-miR-39 in serum. (A) Hemolysis was induced by ultrasonification (US) of the blood (control without US). Serum was separated from intact blood cells as described, 2.8 × 106 copies of cel-miR-39 per µL serum were spiked in and serum was incubated for periods of 0, 1, 5 or 24 h. RNA was isolated and miR-122 and cel-miR-39 were quantified by reverse transcription real-time PCR. The figure is representative of three independent experiments. (B) Lysate from 1.3 × 106 erythrocytes (in 600 µL) was added to 300 µL serum and 2.8 × 106 copies of cel-miR-39 per µL serum were spiked-in before incubation at room temperature for 0 and 24 h. RNA was isolated and miR-122 and cel-miR-39 were quantified. miRNA levels after 24 h incubation were normalized to the initial value at timepoint 0. Shown are means + SD of five independent experiments. Statistical significance was calculated using Wilcoxon-Mann-Whitney-U test. *P < 0.05, **P < 0.01.

Blood cells apparently contain an excess of RI as compared to RNase activity [24]. Therefore, we hypothesized that a release of RI from leaky blood cells may account for the increased stability of serum miR-122 upon hemolysis. To test this hypothesis the effect of hemolysis on the degradation of spike-in cel-miR-39 in serum was assessed. cel-miR-39 was spiked into sera obtained from hemolysed or not hemolysed samples. The samples were incubated for 0, 1, 5 or 24 h at room temperature prior to the extraction of RNA and quantification of miR-122 and cel-miR-39. As shown in Figure 4A, cel-miR-39 rapidly disappeared from the non-hemolysed sera. In contrast, in the hemolysed samples significant levels of cel-miR-39 remained detectable after an incubation of 5 h. However, endogenous serum miR-122 was more stable than the exogenous cel-miR-39 even in the hemolysed samples (Figure 4A). Similar results were obtained, when lysed erythrocytes were incubated with serum for 24 h and spike-in cel-miR-39 and endogenous miR-122 were assessed (Figure 4B). These data indicate that hemolysed samples contained reduced miRNA degrading RNase activity compared to non hemolysed sera.

### Incubation of serum leads to an increase of the portion of vesicle-associated miRNA as compared to non-vesicle-associated miRNA

**Figure 5:**
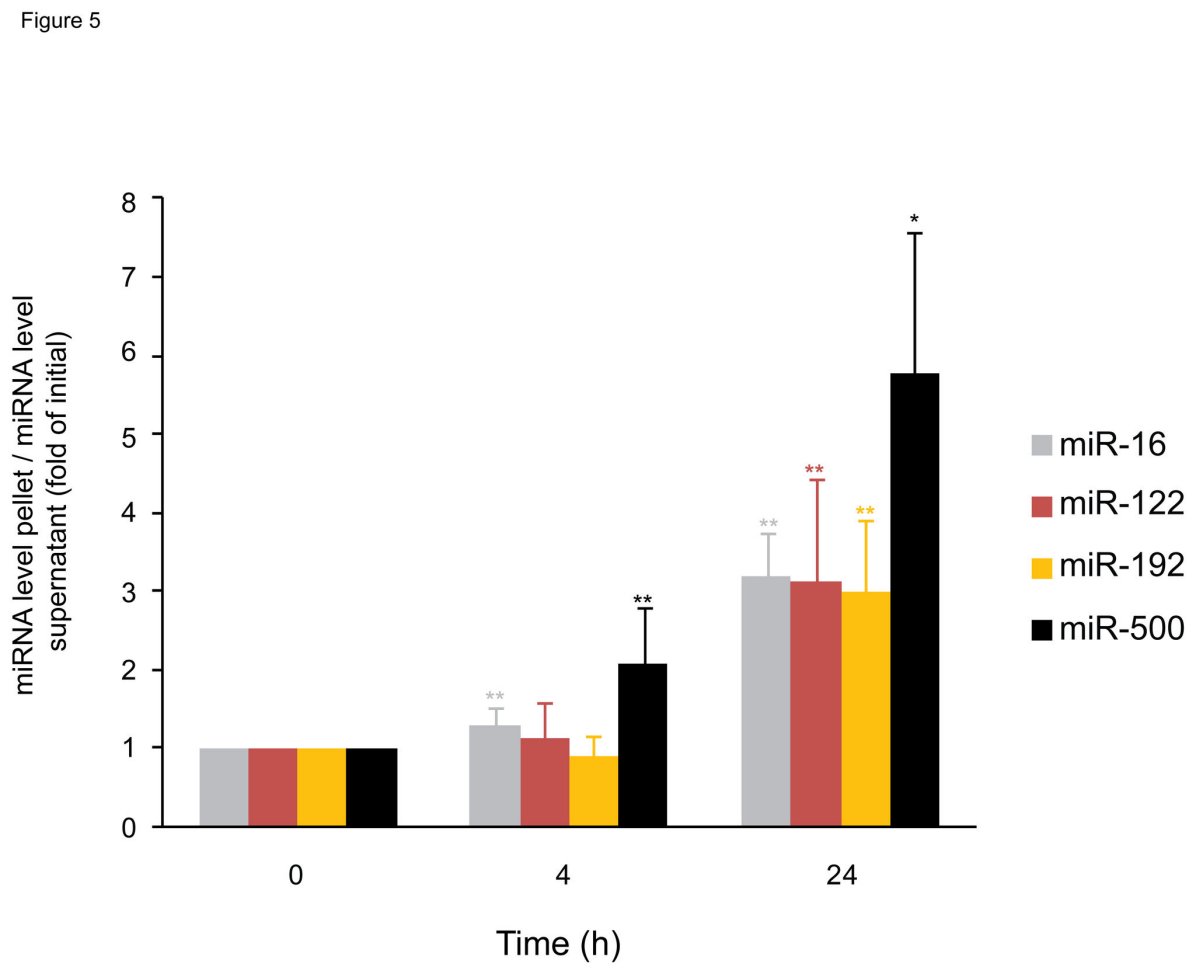
Incubation of serum enriches the portion of vesicular miRNA versus non-vesicular miRNA. Freshly prepared serum (n = 5) was incubated for 0, 4 or 24 h at room temperature before ultracentrifugation to prepare microvesicles. From a 200 µL sample of the supernatant as well as the total vesicle fraction RNA was isolated and miR-16, miR-122, miR-192 and miR-500 levels were determined. Ratios of miRNA levels of the pellet (vesicle) to the supernatant (non-vesicle) fraction were normalized to the initial ratio. Statistically significant differences to the initial value were determined with the Wilcoxon-Mann-Whitney-U test. * P < 0.05, ** P < 0.01.

To investigate if miRNAs associated with vesicles and those not associated with vesicles show different kinetics of decline in serum, sera were incubated for 0, 4 and 24 h at room temperature followed by separation of vesicle- and non-vesicle fractions by centrifugation and determination of miR-16, miR-122, miR-192 and miR-500 in the fractions. As illustrated in Figure 5, the ratio of vesicle- to non-vesicle-associated miRNAs increased upon prolonged incubation of the sera. This suggested that vesicle-associated miRNAs might be more stable in serum than the respective non-vesicle-associated miRNAs. Alternatively, miRNAs may associate with vesicles upon prolonged incubation of the sera.

### Vesicle-associated miRNAs are more resistant to RNase A than non-vesicle-associated miRNAs

**Figure 6:**
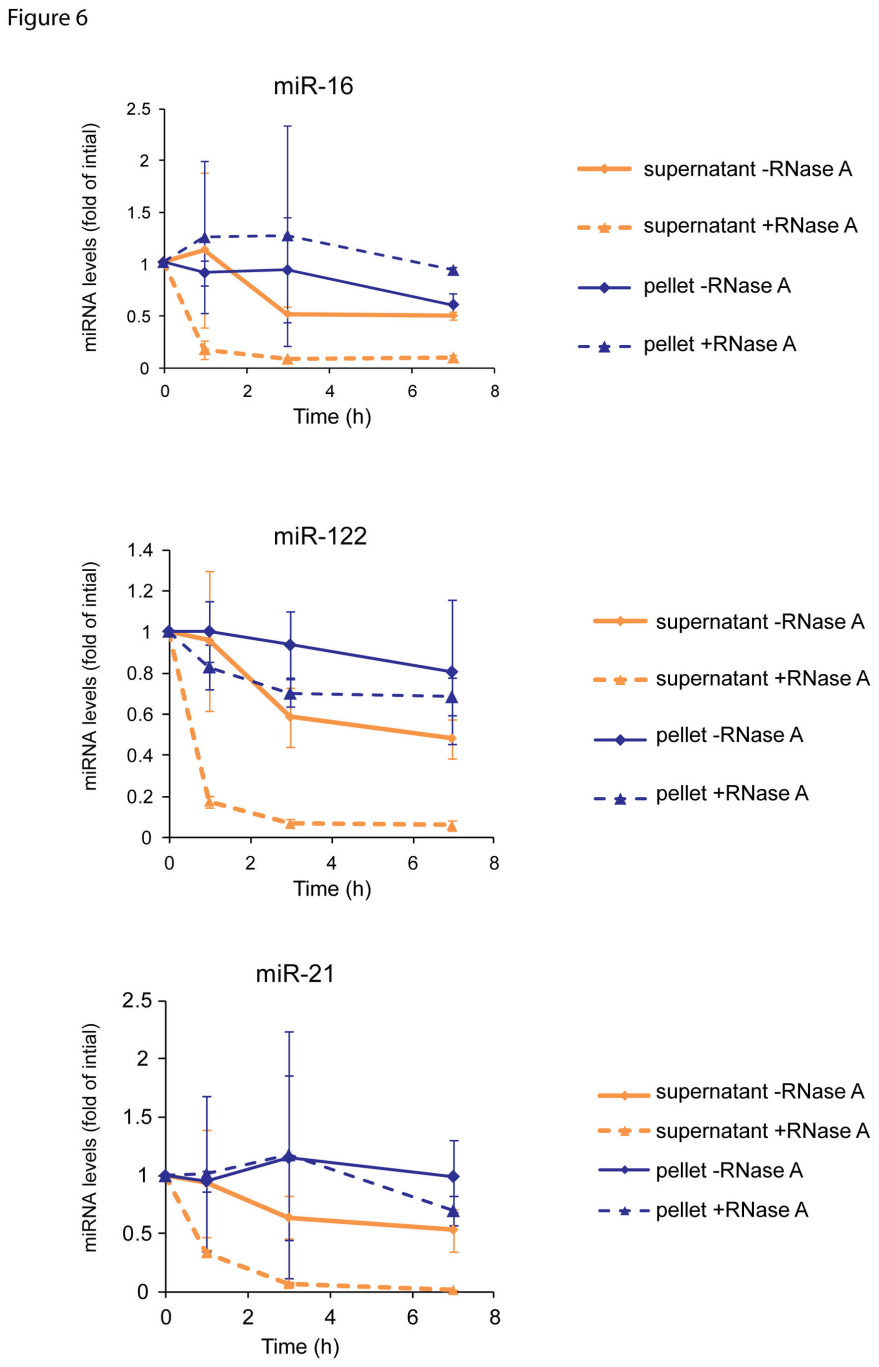
miRNAs encapsulated within vesicles are more resistant to RNase A than non-vesicle-associated miRNAs. Freshly prepared serum (n = 3) was ultracentrifugated to separate the vesicle (pellet) and supernatant fraction prior to incubation at room temperature for 0, 1, 3 or 7 h. After the indicated time RNA was isolated and levels of miR-16, miR-122 and miR-21 analyzed by reverse transcription real-time PCR. Data were normalized to the corresponding values without preincubation. Wilcoxon-Mann-Whitney-U test revealed statistically significant differences between microRNA levels in the supernatant fraction under RNase A treatment compared to the corresponding levels in the pellet fraction (for miR-16 and miR-122: P < 0.05, for miR-21P < 0.01, respectively).

To investigate if vesicle-associated and those not associated with vesicles show different sensitivity towards RNase A, vesicular and non-vesicular fractions were prepared from sera and were treated with RNase A, followed by the determination of the miR-16, miR-21 and miR-122 contents of the fractions. As shown in Figure 6, miR-16, miR-21 and miR-122 associated with the vesicle fraction were considerably more resistant to RNase A treatment than the respective miRNAs not associated with vesicles. Thus, RNase A treatment eliminated a large portion of miRNAs not associated with vesicles, whereas the vesicle-associated miRNAs were less affected by RNase A.

## Discussion

Extracellularly circulating miRNAs have attracted enormous interest during the last years because of their potential role as a newly discovered mode of cell-to-cell communication as well as potential new disease biomarkers. Several studies have found that the levels of serum or plasma miRNAs remained unchanged over several hours or even days, presumably due to the association with lipids/vesicles and/or with RNA-binding proteins such as Ago2 [5-7,9,25]. However, the different miRNAs may show differential stability during prolonged incubation in serum or plasma [6], and the mechanisms of degradation of cell-free miRNAs had been neglected. In the present study we found considerable differences in the stability of endogenous miRNAs in serum upon prolonged incubation (as well as towards RNase A). Thus, prolonged incubation may lead to alterations of the ratios between different miRNAs. This should be considered for instance when choosing internal microRNAs as normalization controls [26].

Another major finding of the present study is that RI prevented the loss of endogenous miRNAs in serum. This indicates that RNase A family enzymes such as human pancreatic RNase, which comprises approximately 70% of RNase activity in human blood serum/plasma [21-23], is the major miRNA degrading activity in serum. This enzymatic activity also degrades small interfering (si) RNAs in serum [27-30]. Thus, despite the relatively high stability of serum or plasma miRNAs, RNase inhibition may provide a more robust preservation of the miRNA patter in serum or plasma, facilitating standardization of the procedures and improving the comparability of the results obtained at different centers.

RI also prevented the loss of miR-122 from whole blood, which was used as marker miRNA because it is not expressed in blood cells and its levels in serum or plasma are therefore not altered by miRNAs released from blood cells. RNase inhibition already in the blood collection system might enable the discovery of diagnostically or functionally important miRNAs that otherwise escape detection due to their rapid degradation. Within these experiments we noted that hemolysis, in particular of erythrocytes, stabilized serum miR-122. Hemolysis also reduced the loss of spike-in cel-miR-39 in serum. Thus, as blood cells contain high amounts of RI [24], release of blood cell-derived RI is likely to account for the reduced degradation of miR-122 and spiked-in cel-miR-39 in hemolysed samples. The inhibitory effect of hemolysis on miR-122 degradation was reversed by exogenous RNase A. This is compatible with the suggestion that the capacity of the endogenous RI in the hemolytic samples was exceeded by the exogenous RNase A. These data indicate that hemolysis offers a convenient and cheap, nevertheless effective, method to preserve the levels of extracellularly circulating miRNAs not expressed in blood cells.

The present study also provides evidence that within one species of miRNA, the vesicle-associated pool appeared to be more stable and more resistant towards RNase A than that not associated with vesicles. Thus, RNase A treatment of serum samples might be useful to differentiate between vesicle-associated and non-vesicle-associated miRNAs. The utilization of RNase A treatment to distinguish between vesicle- and non-vesicle-associated miRNA in serum or plasma would offer several advantages in comparison to ultracentrifugation: it is less expensive, easier to perform, more rapid, can be performed in smaller sample volumes and more samples can be analyzed in parallel and can thus be easily performed in clinical routine practice with large number of samples. RNase A treatment is also faster than the recently introduced exosome precipitation kits [19]. The determination of exosomal and non-exosomal miR-122 has recently been shown to differentiate between inflammatory and toxic liver injury in mice [19]. Thus, RNase A treatment of serum or plasma may disclose information of extracellularly circulating miRNAs exceeding that of the determination of the total miRNA levels.

In summary, we show here that serum miRNAs associated with vesicles appeared to be more stable in serum and more resistant to RNase A treatment than miRNAs not associated with vesicles, and that RI might be highly useful to preserve the pattern of cell-free circulating miRNAs and may enable the discovery of new miRNAs as biomarkers, which normally escape detection because of their rapid degradation. Stabilization of miRNAs not expressed in blood cells such as miR-122 can also be achieved by hemolysis. These findings are highly important for studies with blood-derived miRNAs, in particular for studies using miRNAs as biomarkers.

## Supporting Information

Figure S1 **Enrichment of the exosomal marker CD81 in the pellet fraction isolated from serum.** Freshly prepared serum was separated into pellet and supernatant fraction by ultracentrifugation as described in Materials and Methods. The fractions were analyzed for the exosomal marker CD81 as described recently (20), using Huh-7 lysate as positive control.
(TIF)
