# Supplementary figures and images for: Differential Stability of Cell-Free Circulating microRNAs: Implications for Their Utilization as Biomarkers
Source: PLoS One. 2013 Sep 20;8(9):e75184. doi: 10.1371/journal.pone.0075184 (PMC3779196; doi:10.1371/journal.pone.0075184)

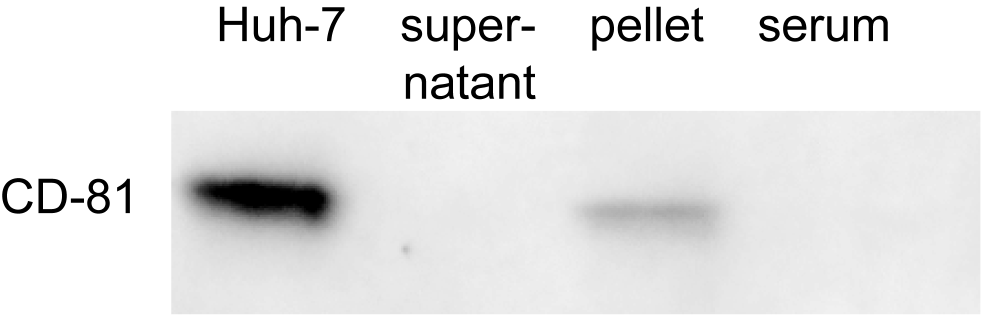

Supplement: Figure S1 — Enrichment of the exosomal marker CD81 in the pellet fraction isolated from serum. Freshly prepared serum was separated into pellet and supernatant fraction by ultracentrifugation as described in Materials and Methods. The fractions were analyzed for the exosomal marker CD81 as described recently (20), using Huh-7 lysate as positive control. (TIF) [file pone.0075184.s001.tif]
